# Intramedullary spinal cord metastases from breast cancer: detection with ^18^F-FDG PET/CT

**DOI:** 10.3332/ecancer.2013.329

**Published:** 2013-07-01

**Authors:** Laura Gilardi, Stefano Vassallo, Marzia Colandrea, Laura Lavinia Travaini, Giovanni Paganelli

**Affiliations:** Division of Nuclear Medicine, European Institute of Oncology, Milan, Italy

**Keywords:** spinal cord metastases, breast cancer, PET/CT, ^18^F-FDG

## Abstract

A 35-year-old woman, already treated with surgery, chemotherapy, and radiotherapy for a ductal carcinoma of the left breast, underwent an ^18^F-fluorodeoxyglucose positron emission tomography/computed tomography (^18^F-FDG PET/CT) scan for an increase of the serum markers carcinoembryonic antigen (CEA) and cancer antigen 15.3 (CA15.3). The scan showed multiple FDG-avid lesions in the liver and bone. The images also detected two areas of uptake in the dorsal and lumbar spinal cord, which were suspicious for metastases; magnetic resonance imaging (MRI) confirmed these lesions.

A 35-year-old woman underwent a left mastectomy in November 2011 and subsequent chemotherapy and radiotherapy for a ductal breast carcinoma. An adjuvant hormonal treatment with tamoxifen and Luteinizing Hormone-Releasing Hormone analogues was then started. In September 2012, a follow-up blood test revealed an increase of the serum markers CEA and CA15.3, so she underwent an ^18^F-FDG PET/CT scan (injected dose: 171 MBq; serum glucose level: 70 mg/dl; time between injection and acquisition: 73 min) that showed multiple FDG-avid lesions in the liver and bone (maximum intensity projection image in Figure 1A). A sagittal PET and fused PET/CT images also detected two areas of focal uptake in the dorsal and lumbar spinal cord (SUVbw max 4.1 and 4.7, respectively; see Figure 1B and C—arrows), which were suspicious for metastases. An MRI confirmed these lesions, which had maximal diameters of 8 and 9 mm (Figure 1D—long arrows), and also detected a 4-mm metastasis (Figure 1D—short arrow), which did not show significant radiotracer uptake due to its limited dimension. In this case, the sensitivity was higher for the MRI than for the PET/CT, but the latter is usually performed from the skull base to the mid-thigh, allowing the detection of distant metastases anywhere in the body, even in unusual sites [1]. Indeed, intramedullary spinal metastatic lesions are exceedingly rare: they account for 3.4–5% of myelopathies in cancer patients and comprise 1–3% of all intramedullary spinal cord tumours. Lung cancer, especially small-cell carcinoma, accounts for the majority of cases (54%). Other common culprits are breast carcinoma (13%), melanoma (9%), lymphoma (5%), and renal cell carcinoma (4%) [2, 3]. The metastatic lesions are usually solitary but may be multifocal in 15% of cases [4]. In three-fourths of reported patients, the time from the onset of neurological symptoms to the development of the full neurological deficit is less than one month [5, 6]. The identification of intramedullary spinal cord metastases in an asymptomatic patient, as occurred in our case, allows treatments to be performed early in the course of the disease, improving the patient’s prognosis and quality of life. Detection of these rare metastases through ^18^F-FDG PET/CT has already been reported in the literature for lung cancer and renal cell carcinoma [7–10]. To the best of our knowledge, this is the first report on spinal cord metastases from breast cancer detected by PET/CT.

## Conflicts of interests

The authors declare that they have no conflicts of interest.

## Figures and Tables

**Figure 1:**
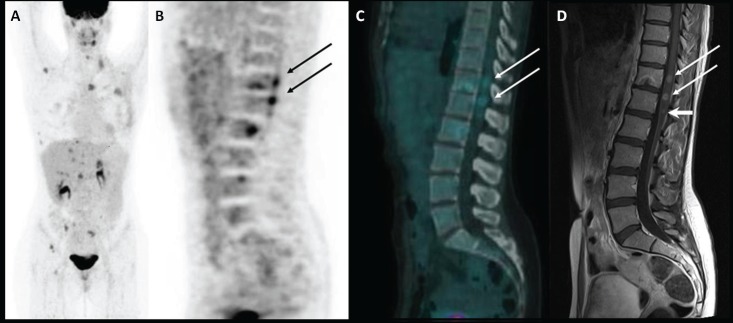
Maximum intensity projection image of 18F-FDG PET/CT scan (A); PET, CT, and MRI sagittal images of spinal cord metastases (B, C, and D respectively).
